# Intestinal mucus components and secretion mechanisms: what we do and do not know

**DOI:** 10.1038/s12276-023-00960-y

**Published:** 2023-04-03

**Authors:** Chunyan Song, Zhenglong Chai, Si Chen, Hui Zhang, Xiaohong Zhang, Yuping Zhou

**Affiliations:** 1 Department of Preventive Medicine, Health Science Center, Ningbo University, Zhejiang Key Laboratory of Pathophysiology, Ningbo, Zhejiang 315211 China; 2 The Affiliated Hospital of Medical School, Ningbo University, Institute of Digestive Disease of Ningbo University, Ningbo, Zhejiang 315020 China

**Keywords:** Ulcerative colitis, Physiology

## Abstract

Damage to the colon mucus barrier, the first line of defense against microorganisms, is an important determinant of intestinal diseases such as inflammatory bowel disease and colorectal cancer, and disorder in extraintestinal organs. The mucus layer has attracted the attention of the scientific community in recent years, and with the discovery of new mucosal components, it has become increasingly clear that the mucosal barrier is a complex system composed of many components. Moreover, certain components are jointly involved in regulating the structure and function of the mucus barrier. Therefore, a comprehensive and systematic understanding of the functional components of the mucus layer is clearly warranted. In this review, we summarize the various functional components of the mucus layer identified thus far and describe their unique roles in shaping mucosal structure and function. Furthermore, we detail the mechanisms underlying mucus secretion, including baseline and stimulated secretion. In our opinion, baseline secretion can be categorized into spontaneous Ca^2+^ oscillation-mediated slow and continuous secretion and stimulated secretion, which is mediated by massive Ca^2+^ influx induced by exogenous stimuli. This review extends the current understanding of the intestinal mucus barrier, with an emphasis on host defense strategies based on fortification of the mucus layer.

## Gut health: Mucus barrier is key to intestinal defences

The intestine’s mucus barrier is a multi-component system that, aided by a continual flow of mucus secretions, helps to protect the gut from pathogens. Xiaohong Zhang, Yuping Zhou and colleagues from Ningbo University, China, review the mucin proteins, antimicrobial peptides and other proteins that collectively determine the structure and function of the thick gel-like mucus that covers the surface of the gut. Specialized secretory cells in the gut lining produce this intestinal mucus, both through a continuous steady baseline process and in response to stress and other stimuli. Impaired secretion or loss of the protective mucus layer can expose intestinal cells to pathogenic microbes that contribute to inflammatory bowel disease, colorectal cancer and other gut disorders. A better understanding of mucosal components and turnover could therefore lead to new therapies for these diseases.

## Introduction

Humans have evolved a three-dimensional mucosal barrier system to maintain local microenvironment homeostasis and systemic health, preventing the invasion of various exogenous antigens and commensal and pathogenic microorganisms into the colonic lumen. The intestinal mucosal barrier is composed of four parts: microbial, mucus, mechanical, and immune barriers^1,2^. As the central component of the mucosal barrier, the mucus layer maintains the homeostasis of intestinal flora by nourishing intestinal symbiotic bacteria and protects the intestinal epithelium against intestinal pathogenic bacteria. The mucus layer also exerts immunological effects by directly binding pathogenic bacterial glycans via lectin-like proteins on immune cells such as those on dendritic cells^3^. Defects in the mucus barrier are associated with intestinal diseases such as inflammatory bowel disease and colon cancer and extraintestinal disorders such as liver disease and diabetes^4^. The continual mucus secretion into the gastrointestinal tract of approximately 10 L per day underlies this protective function^5^. In recent years, significant progress has been made in identifying the components of the mucus layer, understanding their function and identifying the mechanisms underlying mucus secretion. The mucus barrier is garnering increased attention from the scientific community, warranting an overall and accurate overview of mucosal components. In this review, we detail recent findings by highlighting the emerging roles of various mucus components in delineating the stereoscopic structure of the mucus barrier. In addition, because the protective function of mucus is mainly attributable to continuous mucus secretion into the lumen, the mechanism for mucus secretion is elucidated to identify clues into the consolidation of the mucus barrier.

## Mucus barrier impairment is important to colonic inflammation etiology

The colonic mucus layer is a lamellar structure consisting of a dense inner layer and a loose outer layer. The inner layer is attached to the surface of the intestinal epithelium and gradually expands outward to form an outer layer that continuously secretes mucus. Under physiological conditions, the inner layer is mostly sterile, but the outer layer serves as a habitat for microorganisms by providing nutrients and adhesion sites for symbiotic flora. The hierarchical structure of the mucus barrier is key in preventing bacterial penetration and blocking direct contact of bacteria and the intestinal epithelium^6^.

The impairment or loss of the protective mucus layer may cause direct exposure of the intestinal epithelium to microorganisms or pathogens, thereby triggering the development of specific diseases^4^. For instance, penetrating the intestinal mucus barrier is critical for pathogens such as bacteria or worms to induce intestinal symptoms. Diarrhea caused by amoebae is closely related to their attachment to the mucus layer via the release of Gal-lectin as well as by opening a channel for their invasion of the intestinal epithelium induced by mucus decomposition mediated by specific proteases^7,8^. Increased permeability of the mucus barrier paves the way for the occurrence and development of ulcerative colitis (UC)^9^. Mice with dextran sodium sulfate (DSS)-induced colitis show significantly increased permeability of the mucus layer not decreased layer thickness, resulting in direct contact of intestinal microorganisms with the intestinal epithelium, causing inflammation^4^. Patients with UC present with an abnormally high proportion of sulfate-reducing bacteria in the gut, and these bacteria reduce sulfate to hydrogen sulfide by breaking the disulfide bonds between mucin 2 (MUC2) and thus increase the permeability of the mucus layer^10^. Irritable bowel syndrome in a Wistar rat model of water-avoidance stress was linked to a mucin O-glycosylation disorder, which caused the flattening of the mucus layer and loss of its cohesive properties as well as increased intestinal permeability^11^.

## Functional components of the mucus layer

**Fig. 1:**
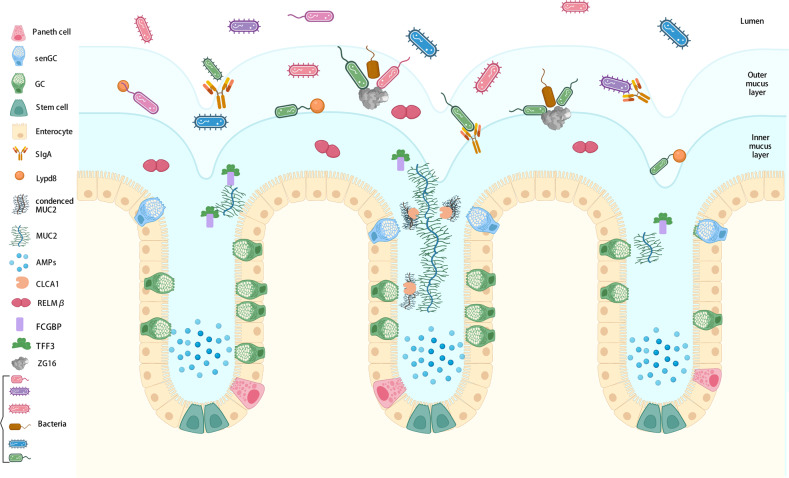
Main components of the intestinal mucus layer. The colonic mucus layer consists of a dense inner layer and a loose outer layer. Multiple components are involved in the maintenance of the structure and function of the mucus barrier in addition to MUC2, which composes the skeleton of the mucus layer. FCGBP and TFF3 act synergistically to enhance the mucus barrier and exert antibacterial effects, while the metalloenzyme CLCA1 is involved mainly in the stratification and expansion of mucus. ZG16, RELMβ, Lypd8, sIgA, and AMP exert bacteriostatic or bactericidal effects under different conditions.

The mucus layer is a very hydrated and complex viscoelastic medium, with MUC2 as its skeleton component. MUC2 cooperates with other components to consolidate the structure of the mucosal barrier and regulate local microenvironment homeostasis. Therefore, it is important to comprehensively analyze the complex components of the mucus barrier and their roles in regulating mucus barrier homeostasis and inhibiting intestinal inflammation (Fig. 1).

### Structural skeleton of the mucus barrier: MUC2

To date, 21 mucins have been identified, named from MUC1 through MUC21 according to the order in which they were discovered. They have also been classified into membrane-related mucins and secretory mucins according to their structural characteristics and biological functions. Membrane-associated mucins include MUC1, MUC3A/B, MUC4, MUC12, MUC13, MUC15, MUC17, MUC20, and MUC21. Secretory mucins are categorized into two subclasses: gel-forming and nongel-forming mucins. MUC2, MUC5AC, MUC5B, MUC6, and MUC19 are gel-forming mucins involved in protection, transportation, lubrication, and hydration, and MUC7, MUC8, and MUC9 are nongel-forming mucins^12^.

MUC2 is the predominant component of the colonic mucus layer function as its structural skeleton. It is a macromolecular glycoprotein comprising more than 5,000 amino acids, and its protein skeleton is synthesized in ribosomes. MUC2 is composed of the following conserved domain structures: a D’ domain containing only a trypsin inhibitor-like (TIL) domain and a fibronectin type I-like (E) domain; D1, D2, and D3 domains forming a von Willebrand D (vWD) structure, a C8 module, and TIL and E domains; the first cysteine domain (CysD); a small proline-, threonine- and serine-enriched (PTS) domain, the second CysD domain; a large PTS domain; a D4 domain containing vWC structure; and a cysteine-knot (CK) domain^13^.

MUC2 monomers are linked by disulfide bonds in the endoplasmic reticulum to form oligomers, which leave the endoplasmic reticulum and enter the Golgi. MUC2 monomers cannot enter the Golgi apparatus. Notably, ATP is required for MUC2 oligomer transport to the cis side of the Golgi. MUC2 is glycosylated in the Golgi, with oligosaccharide chains of different lengths linked to the PTS domain on the protein skeleton by O-glycosidase, and eventually, mature MUC2 molecules with molecular weights in the range of megadaltons are formed^14^. These MUC2 proteins are tightly wrapped in mucus secretory granules and stored as vesicles in goblet cells (GCs), which are specialized intestinal epithelial cells. GCs produce and secrete mucus granules into the intestinal lumen where MUC2 polymers rapidly expand, increasing in size by more than 1000-fold through depolymerization and hydration^15^, and unfold to form a large reticular lamellar structure facilitated by the action of HCO_3_^-^^16^. MUC2 lamellae are connected by noncovalent binding of the D3 domains or covalent isopeptide bonds between CysDs^17,18^. The resultant dense mucin network separates bacteria from intestinal epithelial cells and blocks microorganism invasion into intestinal epithelial cells^19^. In contrast, the loose external mucus layer provides a good habitat for symbiotic microorganisms, which consume MUC2-linked glycans as energy sources and thus decompose and ferment them to produce short-chain fatty acids, which are oxidative phosphorylation substrates in intestinal epithelial cells^20^. A significant increase in the number of GCs and MUC2 mRNAs was observed in obese rats fed high-dose soy isoflavone, resulting in increased colonic mucus secretion and a reduced colonic inflammatory response^21^. MUC2 can transmit immunomodulatory signals to dendritic cells, facilitating intestinal mucosa acquisition of immune tolerance. Small intestinal dendritic cells can penetrate mucus pores and bind to MUC2 via the galectin-3-dectin-1-FcgRIIB receptor complex, activating dendritic cell β-catenin and interfering with the expression of inflammatory factors by inhibiting the transcription of nuclear factor κB (NF-κB)^21^.

### Partnership between FCGBP and TFF3

The trefoil factor (TFF) family comprises small-molecule polypeptides secreted mainly by gastrointestinal cells, and their secondary structures are characterized by one or two unique trefoil domains^22^. The TFF family in mammals includes breast cancer-associated pS2 peptide (pS2/TFF1), spasmolytic polypeptide (SP/TFF2), and intestinal trefoil factor (ITF). The intestinal trefoil factor (also known as trefoil factor 3, TFF3), the most recently discovered member of the TFF family, is secreted mainly by intestinal GCs along with MUC2, and in combination with MUC2 and other components, forming the first defensive line of the intestinal barrier. TFF3 is a cysteine-rich secretory peptide predicted to comprise 59 amino acid residues, of which six cysteine residues form three pairs of disulfide bonds, forming a clover-like structure, also known as the P domain. The cleft between loops 2 and 3 in the clover domain forms the binding site for oligosaccharides, such as mucin, or aromatic amino acids^23^. The P domain stabilizes and compresses the structure of TFF3, which may contribute to its proteolytic and acid resistance^24^.

TFF3 promotes the repair of intestinal epithelial injury by regulating the expression of tight junction proteins in intestinal epithelial cells and the resulting intercellular contacts. TFF3 can anchor to E-cadherin on the cytoskeleton to form a cell adhesion junction complex (an E-cadherin/β-catenin complex) by upregulating E-cadherin expression and increasing the E-cadherin and β-catenin connection. The resulting complex mediates the homophilic interaction of E-cadherin on the surface of adjacent cells, promoting intercellular adhesion and thereby facilitating the migration of intestinal epithelial cells to a damaged areas, where they cover the wound for the rapid repair of epithelial injury^25^. During inflammation, TFF3 reduces the paracellular permeability of the intestinal epithelium to protect the intestinal epithelium from invasion by exogenous microorganisms by upregulating the expression of the tight junction protein Claudin-1 and downregulating the expression of the cation channel-forming protein Claudin-2^26^. TFF3 maintains intestinal mucosal integrity not only by promoting intestinal epithelial repair but also by modulating the inflammatory response. Moreover, TFF3 reduced the expression of the LPS-induced proinflammatory cytokines interleukin-8 (IL-8) and IL-6 in HT-29 cells in vitro^27^. In addition, TFF3 induced the production of the decay-accelerating factor (DAF), which protects the intestinal epithelium from autologous complement injury by preventing the assembly of C3 and C5 invertases and the subsequent amplification of activated classical and alternative complement pathways^28^. A recent study showed that TFF3 expression was upregulated in colorectal mucinous carcinoma tissues compared with that in normal tissues, and its elevated expression was associated with advanced colorectal mucinous carcinoma, vascular or nerve invasion, and a poor prognosis^29^.

IgG Fc-binding protein (FCGBP) is also a component of the colon mucus and contains 13 vWDs, 12 cysteine-rich domains, and 12 trypsin inhibitor-like domains. TFF3 exists mainly as a heterodimer, while FCGBP is expressed and secreted by colon GCs as a partner protein linked to TFF3 by disulfide bonds. The initial protein bound to the Fc fragment of IgG in colonic mucus was observed and was subsequently named FCGBP^30^. Although the function of the FCGBP is not fully understood, it has been demonstrated that it as part of innate immune mucosal defense, as FCGBP and TFF3 heterodimers cooperatively inhibit pathogen attachment and enhance microbial clearance in the early stages of microbial infection^31^. The TFF3-FCGBP heterodimer also interacts with MUC2 via covalent and noncovalent bonds to maintain the structural integrity of the mucus barrier^32^. The network structure formed by the noncovalent combination of TFF3 and MUC2 affects the rheological properties of mucus and protects the mucus vesicles released from the apical side of GCs^33^. Dietary intervention consisting of the long-chain polyunsaturated fatty acid eicosapentaenoic acid (EPA) significantly inhibited DSS-induced experimental enteritis in mice by increasing the generation of the mucus component TFF3 and maintaining the intestinal mucus barrier^34^.

### The mucus “shaper”: CLCA1

The calcium-activated chloride channel regulator (CLCA) family of zinc-dependent metalloproteinases shows characteristics of secretory and self-cleaving activation^35,36^. To date, four CLCAs have been identified in humans (hCLCA1–hCLCA4), eight CLCAs have been identified in mice (mClCA1, CLCA3a1, CLCA3a2, CLCA3b, 3 C, 4 A, 4B, and 4 C), four CLCAs have been identified in cattle (bCLCA1, bCLCA2 (LU-ECAM-1), bCLCA3, and bCLCA4), and CLCA has also been identified in pigs and horses^37^. hCLCA1, the first CLCA family member to be identified, is predominantly expressed in the intestinal epithelium, including the mucus layer of the small and large intestine. mCLCA3, originally known as goblet cell protein-5 (GOB-5), was later renamed mCLCA1 because of its high homology with hCLCA1. mCLCA1, mCLCA2, and mCLCA4 were renamed CLCA3a1, CLCA3a2, and CLCA3b, respectively, due to their high homology with human CLCA3^38,39^. Notably a 35 kDa carboxyl terminal segment of the 110 kDa CLCA1 protein undergoes autocleavage in the endoplasmic reticulum and is then glycosylated to form mature CLCA1, which carries most of the original amino terminus and a small portion of the carboxyl terminus and has a molecular weight of 75–90 kDa. There are five highly conserved domains in mature CLCA1: (1) an amino-terminal signal sequence that directs CLCA1 to the secretory pathway; (2) a zinc-dependent metalloproteinase catalytic domain (CAT) with a conserved HExxE motif and a cysteine-rich (Cys) region; (3) a VWA domain with a conserved metal-ion-dependent adhesion site (MIDAS); (4) an unassigned β-sheet-enriched domain (BSR); and (5) a fibronectin type III (FnIII) domain in the C-terminus^13,35,36^. Mature CLCA1 monomers are linked by disulfide bonds to form dimers, which are connected at the amino termini in a noncovalent manner. Several CLCA1 molecules can polymerize to form globular noncovalent oligomers, which are stored and secreted as oligomers^13^.

Secreted CLCA1 activates calcium-activated chloride channels such as transmembrane protein 16 A (TMEM16A), also known as anoctamin 1 (ANO1)^40^, and the subsequent outflow of chloride ions is enhanced by the VWA domain in the amino terminus^41^. Intracellular increases in Ca^2+^ levels significantly enhance the transport of HCO_3_^-^ by TMEM16A/ANO1, resulting in a large amount of HCO_3_^-^ being secreted into the intestinal lumen^42^. The alkaline environment created by HCO_3_^-^ results in loosening of the mucus structure and its unfolding into a reticular structure, forming a gradient mucus layer with decreasing density and increasing permeability from the inner to the outer layer. Thus, newly secreted mucus constantly pushes the previously generated inner layer of mucus toward the lumen, renewing the mucus layer.

Cystic fibrosis transmembrane conductance regulator (CFTR) is involved in the modulation of HCO_3_^-^ secretion. A decreased intracellular Cl^-^ concentration can result in activation of with-no-Lysine kinase (WNK) and phosphorylation of sterile downstream 20/SPS1-related proline/alanine-rich kinase (SPAK) and oxidative stress responsive kinase 1 (OSR1), transforming the CFTR channel function from that of a main Cl^-^ channel to a HCO_3_^-^ channel, which mediates the outflow of HCO_3_^-^ and maintains a suitable alkaline environment^43^. In addition, CLCA1 is a metalloprotease, and its amino terminal CAT, Cys, and VWA domains can cleave the amino terminus of MUC2 into four segments: D’-D3-CysD (125 kDa), GFP (32 kDa), D1-D2 (88 kDa), and D3-CYSD (88 kDa), thereby inducing the structural rearrangement of MUC2 molecules^13^.

CLCA1 is closely associated with mucus-related diseases. CLCA1 can bind to receptors on the surface of respiratory epithelial cells, thereby activating the MAPK13 signaling pathway and inducing mucin gene expression^44^. In addition, IL-13 activates its receptor and downstream signal transducer and activator of transcription 6 (STAT6), which in turn, enhances downstream CLCA1 expression and MUC5AC secretion by airway epithelial cells. Specific knockout of SAM-pointing domain-containing ETS transcription factor (SPDEF) can inhibit the production of MUC5AC in human respiratory epithelial cells, which is induced by IL-13, while suppression of STAT6 blocks IL-13-induced production of SPDEF and MUC5AC^45^. Moreover, when IL-13 is upregulated, SPDEF decreases CLCA1 expression^46^. It is suggested that IL-13 can induce MUC5AC production in human airway epithelial cells through the STAT6/SPDEF signal transduction pathway. Targeted inhibition of the STAT6/SPDEF signaling pathway controls mucus overproduction, which slows the progression of chronic airway inflammatory diseases. Cystic fibrosis (CF) is an autosomal recessive genetic disease that affects multiple organs, such as the respiratory tract, pancreas, intestine, and liver, and is characterized mainly by mucinous gland hyperplasia and viscous secretions. The disorder is associated with reduced channel activity caused by mutations in the CFTR gene and impaired transport of Cl^-^ and HCO_3_^-^ ions, resulting in obstruction by thickened secretions and organ dysfunction^35^. Mucin secretion in the CF colon is dependent on CFTR expression and its associated CLCA1 production^47^. Data obtained from animal experiments revealed that the expression of CLCA1 was decreased in CF mice. Increasing the expression of CLCA1 significantly improved mucus-related symptoms^48^. These results suggest that CLCA1 can ameliorate intestinal mucus thickening and obstruction in cystic fibrosis patients.

Ulcerative colitis (UC) is an inflammatory disease of the colonic mucosa characterized by persistent dysregulation of intestinal epithelial barrier function. Spontaneous loss of CLCA1 protein expression has been found in the colonic epithelium of patients with ulcerative colitis^49^. A genome-wide expression analysis revealed that CLCA1 expression was significantly downregulated in patients with UC^3^. Some researchers have observed DSS-induced colitis in CLCA1^-/-^ mice. The results showed that the CLCA1^-/-^ mice did not show increased colonic inflammatory symptoms or histopathological abnormalities compared with wild-type mice, but the chemokine (C-X-C motif) ligand-1 (CXCL-1) and IL-17 mRNA levels in neutrophils were increased in CLCA1^-/-^ mice^50^. Therefore, the role of CLCA1 in UC remains unclear.

### The sentinel of the mucus layer: ZG16

Zymogen granule protein 16 (ZG16), a 16 kDa soluble protein without a transmembrane domain, was discovered in rat pancreatic zymogen granules^51^. During the formation of rat zymogen granules, ZG16 concentrates secretases into a dense inner core, which is attached to the granular membrane via the interaction of ZG16 with sulfated glycosaminoglycans in the inner granular membrane^52^.

ZG16 is a lectin with a core lectin domain, and the amino acid sequence of its glycosyl recognition region shares 52% homology with the sequence of durian lectin (Jacalin); therefore, ZG16 belongs to the Jacalin lectin family. ZG16 comprises three β-hairpin structures (I: β1, β2, β11, and β12; II: β3, β4, and β6; III: β7-β10), which form the core β-prism fold, and an α-helix structure between the β2 and β3 chains. The glycosyl group-binding site in ZG16 consists of three rings, a GC ring between β1 and β2, a recognition ring between β7 and β8, and a linker ring between β11 and β12. ZG16 can bind to mannose through a sugar-binding site, form a positively charged channel on the surface of the protein molecule through lysine residues at positions 102, 106, and 122, and bind to negatively charged glycosaminoglycans^53^. ZG16 is a protein secreted by serous parotid gland acinar cells, pancreas acinar cells, and colon goblet cells, and is characterized by sulfated glycosaminoglycans combined with mannan^53,54^.

The key feature of exogenous pathogenic microorganisms such as pathogenic Candida and Malassezia is a mannan-covered cell wall, which is also seen in the cell wall of nonpathogenic *Saccharomyces cerevisiae*. ZG16 can specifically bind to mannan on the cell wall surface of these fungi. This binding does not affect the growth of the fungi and their adhesion to intestinal epithelial cells but may send a message to the host immune system and drive an immune response with signaling to resist invading pathogenic microorganisms. The intestinal symbiote *Candida albicans* is also enriched with mannan, to which ZG16 can bind, thereby preventing this fungus from entering the blood through the intestinal wall and causing entheogenic infection^54^. ZG16 does not kill bacteria directly, but combines with peptidoglycans in the cell wall of gram-positive bacteria, forming bacterial aggregates that cannot readily cross the mucus layer or be eliminated from the intestinal epithelium. Since most beneficial intestinal bacteria carry a low peptidoglycan content in their cell wall, ZG16 exerts little effect on beneficial bacteria. Moreover, ZG16^-/-^ mice presented with a distal colon mucus layer of normal thickness, but their mucus layer showed increased permeability, and intestinal bacteria were thus more likely to penetrate the mucus layer and invade the intestinal epithelium compared to their permeability in ZG16^+/+^ mice. In addition, gram-positive bacteria (mainly Firmicutes) have been detected in tail lymph node and spleen tissues of ZG16^-/-^ mice^55^.

The ZG16 protein is absent in colorectal cancer tissues and found at reduced levels in precancerous adenomatous polyps (adenomas) and tissues of chronic ulcerative colitis. Thus, ZG16 expression is successively decreased in normal tissues, adenomas and cancers^56^. Recently, ZG16 expression in colorectal cancer tissues was negatively correlated with the level of programmed death-1 ligand (PD-L1), the degree of distant metastasis, and lymphatic invasion of colorectal cancer. ZG16 overexpression in colon cancer cell lines (the SW480 and HCT116 cell lines) resulted in significantly inhibited proliferation of colon cancer cells due to decreased PD-L1 expression and the enhanced killing effect of NK cells on tumor cells^57^. These results suggest that ZG16 may inhibit tumor cell immune escape and may be a potential target for tumor immunotherapy.

### Dual roles of RELMβ

The resistin-like molecule (RELMS) family was discovered in a mouse model of asthma^58^. To date, four members of the family, RELMα, RELMβ, Resistin, and RELMγ, have been identified. RELM family members carry three domains: an amino-terminal signal peptide, an intermediate variable region of 28~44 amino acid residues, and a relatively conserved carboxyl terminus enriched with 11 cysteine residues; family members share 105~114 amino acid residues and Cys enrichment^59^. RELMβ was first identified in the colonic epithelial cells of mice, and its content was found to be most abundant in the distal colon, followed by the cecum and with a small amount in the ileum^59^. RELMβ is synthesized in goblet cells and secreted into mucus in the form of homodimers. Intestinal RELMβ expression was significantly reduced in mice reared in a sterile environment. However, when germ-free mice were placed in a conventional environment, a large amount of RELMβ protein was synthesized and secreted by goblet cells within 48 h, suggesting that intestinal flora regulated the expression of RELMβ^60^.

RELMβ expression is significantly upregulated during colon inflammation^61^. RELMβ was not involved in the occurrence of enteritis in mice induced by high-dose flagellate infection. Low-dose flagellate infection-induced chronic enteritis in mice resulted in significantly increased interferon-γ (IFN-γ) levels in the intestinal epithelium of wild-type mice, resulting in persistent infection, while the expression of IFN-γ in the intestinal epithelium of RELMβ^-/-^ mice was decreased, and flagellate infection was significantly attenuated^62^. Recombinant RELMβ stimulated the release of the inflammatory factor TNF-α from isolated peripheral blood macrophages. The symptoms of DSS-induced enteritis in RELMβ^-/-^ rats were delayed, and the severity was reduced compared with that in wild-type rats^63^. The antimicrobial activity of RELMβ was evaluated in bacteria grown to the log phase and treated with purified RELMβ, and the results revealed that fewer gram-negative bacteria (*Pseudomonas aeruginosa* and *Citrobacter murine*) survived, while the survival of gram-positive bacteria (Listeria and *Enterococcus faecalis*) was unchanged. These outcomes were explained by the formation of RELMβ channels that penetrate the bacterial cell wall and subsequently kill bacteria^64^. It has recently been shown that RELMβ exhibits antibacterial activity against gram-positive bacteria such as *Staphylococcus aureus*^65^. These studies suggest that RELMβ has bactericidal activity against specific types of bacteria.

Infection of the intestinal tract with Citrobacter resulted in a decreased number of CD4^+^ T cells and reduced IL-22 levels in RELMβ^-/-^ mice, causing impaired epithelial cell proliferation and significantly damaged intestinal mucosa. Treatment of RELMβ^-/-^ mice with recombinant RELMβ protein administered via enema restored the tropism of CD4^+^ T cells to inflammation sites, IL-22 levels in the intestinal epithelium and epithelial cell proliferation, indicating that RELMβ recruited CD4^+^ T lymphocytes and repaired mucosal injury^66^. Thus, RELMβ may play different roles in enteritis with different etiologies.

### The flagellum “holder”: Lypd8

Ly6/plaur domain-containing protein 8 (Lypd8), a member of the Ly6/Plaur family, is a recently discovered antibacterial molecule that was first identified in mouse intestinal epithelial cells. Lypd8 is a highly N-glycosylated phosphatidylinositol (GPI)-anchored protein with 13 asparagine (Asn) residues (at n-glycosylation sites)^67^. Mouse Lypd8 can bind to flagellated bacteria, such as *Proteus mirabilis* (*P. mirabilis*), *Helicobacter mirabilis* (*H*. *mirabilis*) and *Escherichia coli* (*E.Coli*), and inhibit the movement of flagella, separating bacteria from intestinal epithelial cells and thus preventing bacteria from invading the intestinal epithelium of mice. Compared with that in wild-type mice, DSS-induced colonic inflammation was more severe in Lypd8^-/-^mice, and bacterial aggregation was observed in the inner layer of mucus^67^. Colonization of the colon by a large number of Clostridium was observed in Lypd8^-/-^ mice, which resulted in profound proliferation of Th17 cells and neutrophils in the lamina propria and severe colitis^68^. Similar to murine Lypd8, human Lypd8 can bind to flagellated bacteria, such as *P. mirabilis* and *E. coli*, and inhibit their penetration into the mucus layer^69^. Recently, it was confirmed that Lypd8 can directly block the adhesion of Citrobacter to intestinal epithelial cells by binding to tight adhesions on the surface of bacteria and inhibiting the adhesion of bacteria to intestinal epithelial cells^70^. Moreover, Lypd8 expression has been found to be significantly decreased in colon cancer tissues, and the activities of the IL-6/signal transducer and activator of transcription 3 (STAT3) and TNF-α/NF-κB inflammatory signaling pathways were increased compared to those in precancerous and normal tissues^70^. Similarly, the levels of the inflammatory cytokines TNF-α and IL-6 and the phosphorylation of the downstream target proteins NF-κB and STAT3 in human colon cancer cells with Lypd8 overexpressed were significantly decreased, and the proliferation and migration of the cancer cells were inhibited^70^. These results suggest that Lypd8 can be used as a marker and therapeutic target of enteritis and colon cancer.

### The bacteria hunter: sIgA

Secretory immunoglobulin A (sIgA) is an antibody discovered by Tomasi et al. ^71^ in the 1960s and is found in exocrine fluids, such as milk, gastrointestinal fluid, and respiratory tract secretions. sIgA content is highest on the intestinal mucosal surface and is not easily hydrolyzed by nonspecific proteases, making it the primary effector molecule in the mucosal immune system^72^. When antigens contact the intestinal mucosa, antigen recognition cells (M cells) on the mucosal surface recognize and transmit the antigen signal to antigen-presenting cells (such as macrophages, dendritic cells, and lymphocytes), which decompose the antigens into fragments, thereby activating B-lymphoid cells and converting them into IgA^+^ B lymphocytes with the capacity to secrete polymeric immunoglobulin A (pIgA). PIgA binds to the polymeric immunoglobulin receptor (pIgR) on the basal side of mucosal epithelial cells to form the pIgR/pIgA covalent complex, which is taken up by cells via endocytosis. Then, PIgR is cleaved by proteolytic enzymes, and the secretory fragments (SCs) are released and bind with pIgA to form sIgA, which is released into the colonic lumen via exocytosis, while the remaining part is cellularly recycled to generate new pIgR^73^. Moreover, it was found that SC can protect IgA from degradation by host and intestinal microbial proteases in the harsh intestinal environment and enhance adaptive immunity^74^.

When pathogenic microorganisms threaten the intestinal mucosal barrier, sIgA, as the first line of defense in gut-specific immunity, inhibits bacterial movement and adhesion to the intestinal mucosa by capturing the bacteria on the mucosal surface and directly binding to specific sites, thereby preventing bacterial invasion^75^. pIgA can also bind bacteria that have invaded the intestinal epithelium to form immune complexes, some of which is phagocytosed by mononuclear macrophages, with the remaining pIgA–antigen complexes bind pIgR, which is expelled by a pIgR-mediated transmembrane transport mechanism, followed by intestinal peristalsis^75^. Recent studies have shown that the microbiota can regulate the production of sIgA and pIgR in turn, with secretions from the symbiotic microbiota sending signals to intestinal epithelial cells and immune cells, inducing the activation of pattern recognition receptors to promote the production of sIgA^76,77^.

### The bactericidal experts: AMPs

Antimicrobial peptides (AMPs), discovered in 1980^78^, are composed of 20–50 amino acid residues and are enriched with arginine and lysine residues. AMPs constitute a large family with several members that have been identified through a variety of classification methods. Typically, AMPs are classified into four types according to their origin: insect, plant, microbial, and animal. Animal antibacterial peptides include defensins, C-lectins, and cathelicidin family members, and they are derived mainly from neutrophils, epithelial cells, skin secretions, and protein degradation products. The defensin family, which is related to host innate immunity, mounts an important defense against pathogenic bacteria^79^. Defensins can be classified into α-Defensins, β-Defensins, and Ɵ-Defensins according to the position of the cysteine residues and disulfide bonds. C-lectins are mainly regenerative islet-derived proteins (Reg), including Reg1, Reg2, Reg3a, Reg3b, Reg3g, Reg3d, and Reg4. The Cathelicidin family is named for the highly conserved Cathelin peptide^80^. Notably, compared with those in other species, there are few members of the human AMP family, which is composed mainly of defensins, Cathelicidins, Histatins and so on^81^. Human α-defensins can be classified into neutrophil defensins (HNP-1, HNP-3, and HNP-5) and intestinal defensins (HD-5 and HD-6)^82^.

AMPs secreted by Paneth cells, which are located at the base of intestinal crypts, exhibit antiviral and antibacterial properties. AMPs are important components of intestinal innate immunity because of their rapid killing and effective inactivation of pathogenic bacteria. Paneth cells were previously thought to reside only in the small intestine, while the healthy colon was thought to carry neither Paneth cells nor AMPs. Later, it was reported that both metaplastic Paneth cells and AMPs were detected in inflammatory bowel disease (IBD)^83^. AMPs can effectively kill bacteria by binding to negatively charged bacterial membrane lipids through electrostatic attraction mediated by their positively charged surface amino acid residues, thereby forming multiple stable transmural channels and transmembrane ion channels, resulting in exocytosis from bacterial cell bodies and bacterial death due to irreversible damage^84^. AMPs also act as immune regulators by presenting signals to dendritic cells and T cells that activate the immune response^85^. For example, β-defensin by itself can act as a chemokine to drive leukocytes to a site of infection and thus suppress the progression of inflammation and promote mucosal repair^86^. It also attracts immature dendritic cells and CD4 + T cells through the chemokine receptor CCR6, facilitating the maturation and activation of T cells^87^. In addition, Reg3 selectively binds peptidoglycans on the surface of bacteria^88^, including gram-positive bacteria and some gram-negative bacteria, such as *Salmonella typhimurium* and *Pseudomonas aeruginosa*, thereby causing destroying the bacterial cytoderm, causing cytoplasm leakage, and resulting in bacteria death^89^.

## Regulatory mechanisms of mucus secretion

Continual mucus secretion is a key factor in determining the structure and function of the mucus barrier. The mucus barrier is not a static physical barrier, with the inner mucus layer in murine distal colonic tissue found to be renewed every 1–2 hours^4^. It has been estimated that mucus grows spontaneously at a rate of approximately 240 μm/h in humans and 100 μm/h in mice^90,91^. Notably, mucin granule exocytosis is a Ca^2+^-regulated process; therefore, mucus secretion can be classified into two modes (baseline secretion and stimulated secretion) according to the involvement of calcium influx. Baseline secretion is spontaneous Ca^2+^ oscillation-mediated slow and continuous secretion, while stimulated secretion is mediated mainly by substantive Ca^2+^ entry induced by exogenous stimuli. In the colon, surface GCs are critical for baseline secretion, while GCs in the upper part of colonic crypts are critical to mucus secretion in response to stress stimuli. In our opinion, stimulated mucus secretion can be further classified into constitutive secretion mediated by physiological ATP levels and mechanical stimuli and impulsive mucus secretion mediated by strong external inflammatory stimuli. Recently, mucus was shown to be released by baseline secretion in quantities that were several fold greater higher than that released via the stimulated secretion process, although baseline mucus secretion involves the continuous release of mucins at a low rate^92^.

### Spontaneous Ca^2+^ oscillation-mediated baseline mucus secretion

**Fig. 2:**
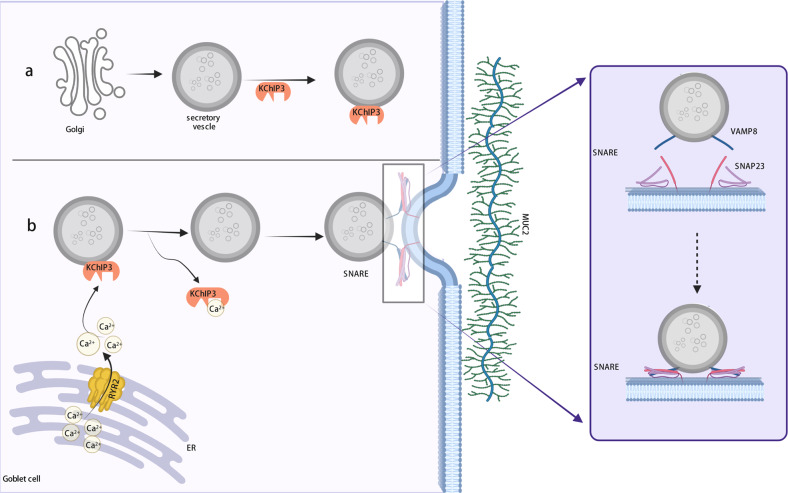
Baseline mucus secretion and SNARE assembly. Baseline mucus secretion is the continuous release of mucins at a low rate. **a** The Golgi apparatus releases mature, primed mucin secretory vesicles filled with MUC2. The high-affinity Ca^2+^ sensor KChIP3, which senses Ca^2+^ concentrations <1 μM, binds to secretory vesicles and prevents them from fusing with the cell membrane in the absence of intracellular Ca^2+^ oscillations as a tonic brake. **b** Intracellular Ca^2+^ oscillation plays an important role in baseline mucin secretion. Spontaneous oscillations in Ca^2+^ from internal stores (mainly in the ER) initiate steady, moderated mucus release in a ryanodine receptor 2 (RYR2)-dependent manner.

Under physiological conditions, colonic GCs continuously synthesize and release mucin to renew the mucus layer and maintain its thickness and physicochemical properties. During the migration of GCs from the bottom of the colonic crypt to the crypt luminal opening, highly ordered vertical microtubules and microfilaments are formed in the cells, and secretory granules are transferred to the top of goblet cells in an orderly manner through the interaction of microtubules, microfilaments, and actin. This group of microtubules and microfilaments separates the secretory granules from other cytoplasmic cellular components and gives the goblet cell its distinctive “goblet” shape. Mucins in GCs are packaged to form secretory granules after polymerization in the endoplasmic reticulum (ER) and glycosylation modification in the Golgi apparatus. In summary, the process of mucus secretion involves the migration of mucus vesicles along the cytoskeleton to the apical side of a cell, the fusion of a vesicle membrane with the cell membrane, and exocytosis (Fig. 2a).

Calcium ions are among of the most abundant cations in the body and are important second messengers in cell signal transduction. Mucus secretion is a calcium-dependent biological process. Intracellular Ca^2+^ stores (principally in the ER) are the sources of Ca^2+^ oscillations in goblet cells. Recently, intracellular spontaneous Ca^2+^ oscillations was confirmed to be key factors regulating mucus secretion under baseline conditions. The ryanodine receptor (RYR) is the largest known high-throughput calcium channel protein and is involved in the generation and maintenance of these spontaneous oscillations. RYR2 mediates the release of Ca^2+^ from the ER, resulting in an increase in the Ca^2+^ concentration in the regions adjacent to the ER. Inositol The 1-,4-,5-triphosphate (IP3) receptor (IP3R) also mediates Ca^2+^ release from the ER. KChIP3 (potassium voltage-gated channel-interacting protein 3), also named DREAM and Calsenilin, is a member of the neuronal Ca^2+^ sensor protein (NCS) family and is a multifunctional Ca^2+^-binding protein with a molecular weight of 29 kDa. KChIP3 is a high-affinity calcium receptor that can sense intracellular Ca^2+^ levels at concentrations lower than 1 µM. KChIP3 has been shown to be localized in a pool of mucin secretory granules and act as a negative regulator of baseline mucin secretion by binding mucin granules and inhibiting mucus release. The binding of Ca^2+^ to KChIP3 followed by KChIP3 dissociation from mature secretory granules allows the fusion of mucin granules with the apical plasma membrane and the subsequent release of mucin into the intestinal lumen (Fig. 2b). Moreover, the KChIP3-related mucus secretion mechanism is not tissue-specific and is conserved in GCs secreting mucin^93^.

Mucus secretion requires large amounts of ATP, making it highly dependent on mitochondrial oxidative phosphorylation for sufficient energy^94^. Commensal bacteria in the intestine can ferment dietary fiber with glycosidase to release short-chain fatty acids. As the main energy substrate in epithelial cells, butyric acid is consumed by intestinal epithelial cells to generate ATP through β-oxidation and thus fuels mucus secretion^95^. In the absence of dietary fiber, glycosidase is leveraged by symbiotic bacteria to produce sugar groups in mucus. In the absence of dietary fiber, mucophilic bacteria degrade mucus, leading to reduced thickness of the colonic mucus layer and destruction of the intestinal barrier, increasing the risk of inflammatory bowel disease^96^. When the expression level of Mir-124-3p in the colon of aged mice was significantly increased, the expression of the O-glycosylation rate-limiting enzyme T-synthetase was reduced, which inhibited the O-glycosylation of mucin. As a result, the permeability of the mucus layer is increased, reducing the barrier to pathogens and bacteria penetration of the mucus layer and their infiltration into the intestinal epithelium, causing colitis^97^. Moreover, Mir-1-3p downregulated the expression of T-synthetase and cooperated with Mir-124-3p to inhibit the effect of T-synthetase, destroying the colonic mucus barrier and increasing the severity of mouse colitis^97^. A recent study demonstrated that the expression of mitochondrial oxidative phosphorylation complexes is decreased in colonic epithelial cells of elderly individuals, and the intestinal mucosa was thus vulnerable to damage and inflammatory bowel disease due to insufficient energy production and decreased mucus synthesis and secretion^11^.

### Stimulated mucus secretion

Stimulated mucus secretion can be classified into constitutive and impulsive secretion depending on the property of the stimulus that induces the mucus secretion.

#### Constitutive mucus secretion

**Fig. 3:**
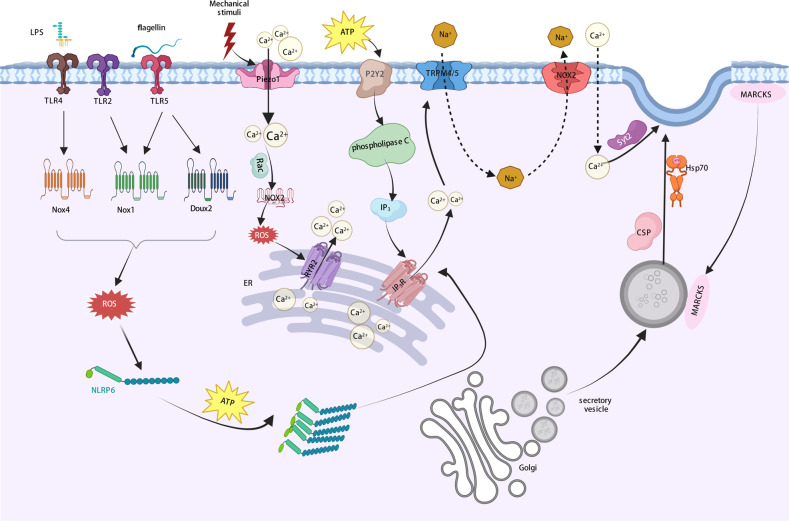
The underlying mechanism for stimulated mucus secretion. Several physiological or pathological stimuli result in a marked increase in intracellular Ca^2+^-triggered stimulated mucus secretion. Intracellular Ca^2+^ levels are increased through two mechanisms. After stimulation with an exogenous stimulus, TRPM4/5 are activated and cooperate with NCX2 to enhance Ca^2+^ influx. LPS and flagella in bacteria induce TLR-mediated ROS production and activate NLRP6, which in turn promotes Ca^2+^ release from the ER. Protein kinase C induces detachment from the membrane and subsequent recruitment of MARCKS onto mucus vesicles, and MARCKS-bound mucus vesicles then migrate to the apical side of cells under the action of Hsp70 and CSP. The low-affinity Ca^2+^ sensor Syt2, which can only sense calcium concentrations greater than 10 μM, senses elevated Ca^2+^ levels and drives the fusion of mucus vesicles with the cell membrane.

Under physiological conditions, the pulling effect of mechanical stimulation induced by intestinal peristalsis and the shear stimulation produced by fluid flow in the intestine are important factors in promoting mucus secretion^98^. The transmembrane cation-selective mechanosensitive ion channel protein Piezo is a mechanosensitive ion channel protein that induces the depolarization and activation of voltage-gated L-type calcium channels, resulting in a further increase in intracellular Ca^2+^, presumably leading to exocytosis^99^. It has been reported that Piezo 1 and Piezo 2 are expressed abundantly in the colon^100^. Moreover, Piezo1-triggered calcium influx was found to induce the activation of microtubules in cardiomyocytes under the action of Rac1 (a calcium-dependent small GTPase), prompting homeostatic ROS production and RYR2 activation by stimulating NADPH oxidase 2 (NOX2)^101^. RYR2 is necessary for Ca^2+^ release from the ER, and ROS-mediated posttranslational modifications increase the sensitivity of RYR2. Piezo-mediated exocytosis may be relevant to the intracellular calcium oscillation triggered by the influx of extracellular calcium and subsequent intracellular calcium release (Fig. 3). Notably, lipopolysaccharide (LPS) from the gram-negative bacterial cell wall increased mucin mRNA expression and promoted mucin secretion in HT-29 MTX cells^102^, suggesting that LPS from commensal bacteria might be a regulatory factor in mucus secretion, especially in the constitutive secretion of mucus.

When stimulated by intense extracellular stimulation, mainly paracrine release of ATP, G protein-coupled receptors (e.g., P2Y2) activate phospholipase C to produce diacylglycerol, which activates protein kinase C (PKC) and IP3. IP3 may induce a rapid burst of calcium release from the ER followed by the activation of calcium-activated monovalent cationic channels in cell membranes: transient receptor potential cationic channel subfamily M member 4 (TRPM4) and its homologous protein TRPM5. TRPM4 and TRPM5 mediate sodium influx and are inactive under physiological conditions. Upon activation by extracellular stimulation, TRPM4 and TRPM5 mediate sodium transport into the cytoplasm, increasing the sodium concentration in areas adjacent to Na + /Ca^2+^ exchanger 2 (NCX2), trigging NCX2 pumping of calcium into the cell when operating in reverse mode. The increase in local calcium concentration attracts a low-affinity calcium sensor, synaptotagmin 2 (Syt2), which only senses only Ca^2+^ concentrations higher than 10 μM and induces the fusion of mucus vesicles with the cytoplasmic membrane^103,104^. In addition, activated PKC phosphorylates myristoylated alanine-rich C kinase substrate (MARCKS) on the cytoplasmic side of the plasma membrane, allowing the separation of MARCKS from the plasma membrane and recruitment to mucin vesicles. MARCKS-bound mucus vesicles then migrate through actin-associated pathways and anchor to the apical side of the cell membrane under the mediation of heat shock protein 70 (Hsp70) and cysteine string protein (CSP) (Fig. 3).

#### Impulsive mucus secretion modulation

Recent studies have demonstrated an impulsive mucus secretion mode. In response to acute inflammatory stimuli, such as invasion of a large number of bacteria into the intestinal epithelium, GCs initiate a massive release response (also known as compound exocytosis), in which most of the mucus granules in cells are released after fusion, with mucus largely eliminated from cells^105^. Some GCs at the opening of colon crypts were observed and named “sentinel goblet cells” (senGCs) since they can express pattern recognition receptor toll-like receptors (TLRs) and recognize pathogen-associated molecular patterns (PAMPs), such as specific structures of bacteria, viruses, and fungi. For example, TLR2 and TLR4 recognize LPS, and TLR5 recognizes flagellin. The TLRs activated by microorganisms by after endocytosis activate their receptors, such as Toll receptor domain-containing adaptor-inducing IFN-β (TLR-TRIF) and myeloid differentiation factor 88 (MyD88)^106^, and subsequently activate downstream Nox/Duox. For instance, ROS synthesis was enhanced after TLR5-induced activation of Nox1 and Duox2, TLR2-mediated Nox1 activation, and TLR4-triggered activation of Nox4^107^. NOD-like receptor family pyrin domain-containing 6 protein (NLRP6) inflammasome activation occurs downstream of endocytosis-dependent ROS synthesis. NLRP6 is highly expressed in the intestinal epithelium and is specifically concentrated in the apical mucosal region, and its deficiency leads to defective mucus granule exocytosis^108^. Moreover, autophagy processes have been demonstrated to be required for proper secretion of mucus granules, as indicated by NLRP6-deficient epithelium lacking clear autophagosome formation^108^. Later, autophagy-induced regulation of goblet cell secretory functions was shown to involve downstream reactive oxygen species signaling. Autophagosomes with LC3 fuse to endosomes containing NADPH oxidases, thereby producing amphisomes, which are required for the maximal production of reactive oxygen species (ROS) derived from NADPH oxidases. Moreover, intracellular calcium mediates the effect of ROS on mucin granule release in colonic goblet cells^109^.

NLRP6 is suppressed under resting conditions and activated upon infection with virus or gram-positive bacteria and is subsequently recruited by apoptosis -associated speck-like protein containing caspase-recruitment domain (ASC) and caspase-1/caspase-11 precursors to be part of the NLRP6 inflammasome. The assembly of the NLRP6 inflammasome involves two polymerization steps. First, oligomeric NLRP6 provides a platform for ASC recruitment through its interaction with the pyrimidine domain (PYD) and induction of ASC polymerization. ASC then recruits caspase-1 via interactions with the caspase recruitment domain (CARD), leading to the activation of caspase-1^110^. In addition, NLRP6 recognizes LPS from gram-negative bacteria through leucine-rich repeats (LRRs) in its C-terminus and then undergoes a conformational change followed by the formation of a linear dimer. In the presence of ATP, NLRP6 homodimers self-assemble into larger oligomers, providing a platform for the recruitment and polymerization of ASC and Caspase-1 as well as the formation of the NLRP6 inflammasome^111^. The NLRP6 inflammasome then triggers the release of Ca^2+^ from the ER and promotes compound Ca^2+^-dependent mucin exocytosis, thereby generating intercellular gap junction calcium signals that induce mucus secretion from adjacent GCs near senGCs in the upper part of the crypt, clearing bacteria at the mouth of the crypt and protecting the lower part of the crypt and intestinal stem cells from bacterial invasion. Finally, after nearly all the mucus is secreted, senGCs fall off^106^. Hence, in response to external stimuli, mucus secretion by senGCs is rapid and impulsive. Mucin release from adjacent GCs near senGCs also depends on NLRP6 inflammasome activation but does not involve endocytosis. Ca^2+^ influx and release from intracellular store in the ER are required for LPS-TLR-ligand-induced secretion, and Ca^2+^ influx plays a role upstream of Ca^2+^ store release (Fig. 3). Moreover, senGCs are less important to the normal mucus barrier, as indicated by mouse strains that cannot trigger senGC activation presenting with a functional inner mucus layer^98,106^.

Notably, the intestinal epithelium increases mucin synthesis to antagonize microbial invasion. Serum amyloid (SAA) 3 protein and TNF-α cooperate to promote MUC2 production and protect epithelial cells from bacterial invasion, while SAA1 and SAA2 may also continuously stimulate MUC2 mucin production^112^. Levels of serum amyloid A3 (SAA3) mRNA in CMT-93 mouse colon epithelial cells were elevated by dead *E. coli* together with LPS, while SAA1/2 mRNA expression was not induced. Moreover, recombinant murine SAA 1 (rSAA1) and 3 (rSAA3) significantly upregulated MUC2 mRNA levels in CMT-93 cells. The mRNA of the inflammatory cytokine TNF-α was significantly increased by rSAA3 intervention in CMT-93 cells^107^. TNF-α has also been shown to induce SAA3 expression in CMT-93 cells^113^ and MUC2 mRNA expression in HT-29 human colonic epithelial cells^114^. It has been postulated that SAA1/2 may continuously stimulate MUC2 mucin production, while the SAA3 protein and TNF-α cooperate to promote MUC2 production and protect epithelial cells from bacterial invasion^112^.

### SNARE complex formation is the key molecular event in exocytotic mucus release

Soluble N-ethylmaleimide-sensitive factor attachment protein receptors (SNARE) are critical for the fusion between mucus vesicles and the plasma membrane during baseline and stimulated mucus secretion. SNARE proteins are located in vesicles and plasma membranes and are connected to each other to form SNARE complexes, with vesicle-associated membrane protein 8 (VAMP8) on vesicle particles, and synaptosomal-associated protein 23 (SNAP23) and syntaxin (Stx) on the plasma membrane. During exocytosis, the scaffold protein Munc18b mediates SNARE complex assembly, and the GTPases Rab and Munc13 play auxiliary roles (Fig. 2). The assembly and formation of the SNARE complex promote the fusion of mucus particles and the cell membrane, leading to the subsequent formation of fusion pores through which mucins are expelled from cells^103,115–117^.

## Conclusion

Multiple functional components of the mucus layer, including the main skeletal component MUC2 and other components, such as TFF3, FCGBP, and CLCA1, are gradually being discovered because of their role in building and consolidating mucus structures or their bacteriostatic or bactericidal functions. Their harmonious cooperation is indispensable for the homeostatic maintenance of the structure and function of the mucus layer, which is pivotal to block intestinal and extraintestinal diseases. The continual secretion of mucus is also a key mechanism for the homeostasis of the mucus layer structure. There are two models of mucus secretion: baseline secretion and stimulated secretion, which are differentiated on the basis of calcium influx levels. Both models are key mechanisms evolved to protect host intestinal epithelium from insults. Notably, spontaneous Ca^2+^ oscillation-mediated slow and continuous baseline mucin secretion is more important for mucus barrier integrity than stimulated secretion. This review is the first to thoroughly delineate the roles of various functional components in shaping the structure and function of the colonic mucus barrier and reveal novel insights into the regulatory mechanisms of mucus secretion, providing clues to develop strategies to strengthen the mucus barrier and maintain intestinal microenvironment homeostasis.
